# CFM-ID 4.0 – a web server for accurate MS-based metabolite identification

**DOI:** 10.1093/nar/gkac383

**Published:** 2022-05-24

**Authors:** Fei Wang, Dana Allen, Siyang Tian, Eponine Oler, Vasuk Gautam, Russell Greiner, Thomas O Metz, David S Wishart

**Affiliations:** Department of Computing Science, University of Alberta, Edmonton, AB, T6G 2E8, Canada; Department of Biological Sciences, University of Alberta, Edmonton, AB, T6G 2E9, Canada; Department of Biological Sciences, University of Alberta, Edmonton, AB, T6G 2E9, Canada; Department of Biological Sciences, University of Alberta, Edmonton, AB, T6G 2E9, Canada; Department of Biological Sciences, University of Alberta, Edmonton, AB, T6G 2E9, Canada; Department of Computing Science, University of Alberta, Edmonton, AB, T6G 2E8, Canada; Alberta Machine Intelligence Institute, University of Alberta, Edmonton, AB, T6G 2E8, Canada; Biological Sciences Division, Pacific Northwest National Laboratory, Richland, WA 99352, USA; Department of Computing Science, University of Alberta, Edmonton, AB, T6G 2E8, Canada; Department of Biological Sciences, University of Alberta, Edmonton, AB, T6G 2E9, Canada; Department of Laboratory Medicine and Pathology, University of Alberta, Edmonton, AB, T6G 2B7, Canada; Faculty of Pharmacy and Pharmaceutical Sciences, University of Alberta, Edmonton, AB, T6G 2H7, Canada; Biological Sciences Division, Pacific Northwest National Laboratory, Richland, WA 99352, USA

## Abstract

The CFM-ID 4.0 web server (https://cfmid.wishartlab.com) is an online tool for predicting, annotating and interpreting tandem mass (MS/MS) spectra of small molecules. It is specifically designed to assist researchers pursuing studies in metabolomics, exposomics and analytical chemistry. More specifically, CFM-ID 4.0 supports the: 1) prediction of electrospray ionization quadrupole time-of-flight tandem mass spectra (ESI-QTOF-MS/MS) for small molecules over multiple collision energies (10 eV, 20 eV, and 40 eV); 2) annotation of ESI-QTOF-MS/MS spectra given the structure of the compound; and 3) identification of a small molecule that generated a given ESI-QTOF-MS/MS spectrum at one or more collision energies. The CFM-ID 4.0 web server makes use of a substantially improved MS fragmentation algorithm, a much larger database of experimental *and in silico* predicted MS/MS spectra and improved scoring methods to offer more accurate MS/MS spectral prediction and MS/MS-based compound identification. Compared to earlier versions of CFM-ID, this new version has an MS/MS spectral prediction performance that is ∼22% better and a compound identification accuracy that is ∼35% better on a standard (CASMI 2016) testing dataset. CFM-ID 4.0 also features a neutral loss function that allows users to identify similar or substituent compounds where no match can be found using CFM-ID’s regular MS/MS-to-compound identification utility. Finally, the CFM-ID 4.0 web server now offers a much more refined user interface that is easier to use, supports molecular formula identification (from MS/MS data), provides more interactively viewable data (including proposed fragment ion structures) and displays MS mirror plots for comparing predicted with observed MS/MS spectra. These improvements should make CFM-ID 4.0 much more useful to the community and should make small molecule identification much easier, faster, and more accurate.

## Graphical Abstract

**Graphical Abstract:**
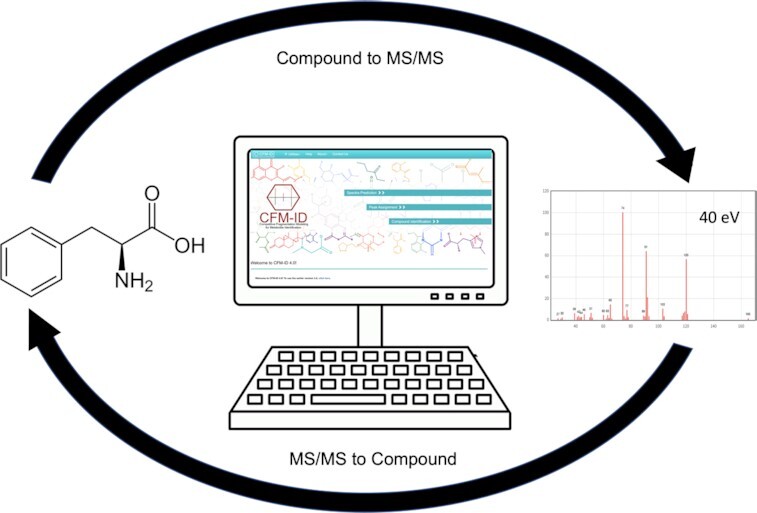
Illustration of the two main functions supported by CFM-ID 4.0. Predicting MS/MS spectra from chemical structures (top) and predicting chemical structures from MS/MS spectra (bottom).

## INTRODUCTION

Electrospray tandem mass spectrometry (ESI-MS/MS) has become the technology of choice for both targeted and untargeted metabolomics studies (1,2). Increasingly, it has also become the preferred technology for identifying small molecules in drug metabolism studies, in exposomics studies, in environmental monitoring, in natural products research and in food science studies (3–5). However, there are two main challenges when using ESI-MS/MS to perform small molecule identification. First, the manual comparison and interpretation of MS/MS spectra is notoriously tedious, time-consuming and error prone. Second, compound identification by ESI-MS/MS requires the existence of a large library of experimentally collected MS/MS spectra, spanning multiple collision energies and multiple platforms, to enable proper spectral matching. Unfortunately, most compounds that are of interest to those working in metabolomics, exposomics or natural products research do not have any experimentally collected MS/MS spectra. For example, the Human Metabolome Database (HMDB) 5.0 (6) has 253,244 metabolites, but only 4,424 of these compounds have experimentally collected ESI-MS/MS spectra (as gathered from multiple internally-collected and open-access MS/MS resources). While several open-access MS/MS spectral databases do exist, such as MoNA (https://mona.fiehnlab.ucdavis.edu/), MassBank EU/Japan (7,8) and GNPS (9), these databases are heavily weighted towards MS/MS data collected on less biologically relevant, less expensive commercial chemicals. Therefore, they tend to cover only a fraction (often < 5%) of known natural products, known environmental exposure molecules, or known drugs. Larger, commercially accessible ES-MS/MS spectral libraries do exist, such as those from NIST (10–12) or METLIN/Bruker (13,14), but these are either very expensive and/or they place restrictions on sales to metabolomics and exposomics researchers. Similar issues also exist with their relatively limited coverage of biologically relevant molecules.

Given that there are literally millions of known metabolites, natural products, food compounds and exposure chemicals (6,15–18) and perhaps 10’s of millions more unknown compounds (19,20), it is unlikely that enough experimental MS/MS spectral will ever be collected to address this central shortcoming of ESI-MS/MS-based compound identification. As a result, more researchers are turning towards *in silico* methods. *In-silico* MS-based compound identification methods were developed to help researchers identify compounds from an experimentally collected MS/MS spectrum without directly needing or querying an experimentally collected reference MS/MS spectral database. State-of-the-art methods for *in silico* MS-based compound identification use a wide array of different techniques, ranging from MS/MS spectral prediction to MS/MS spectral fingerprint analysis. Nearly all of these methods employ combinations of rule-based expert systems and the latest deep learning methods (21–31).

CFM-ID (which stands for Competitive Fragment Modeling IDentification) is an example of an *in silico* MS-based compound identification tool. It was first described in 2014 (28,33). Unlike chemical fingerprint methods, such as SIRIUS 4 (24) and CSI:FingerID (32), CFM-ID uses the latest developments in machine learning to learn, from a small training set of experimental MS/MS spectra and their associated structures, how small molecules will fragment when injected into a quadrupole time-of-flight (QTOF) ESI-MS/MS instrument with collision-induced dissociation (CID) (33). This training/learning process allows CFM-ID to not only predict ESI-MS/MS spectra from a chemical structure, but also to annotate each peak in the predicted spectrum with a probable fragment ion structure. By running CFM-ID through all known small molecule structures (including predicted structures) it is also possible to create a synthetic, *in silico* MS/MS spectral library that is many times larger than any experimentally collected MS/MS spectral library. This *in silico* MS/MS spectral library can then be used to identify compounds by finding matches to experimentally acquired MS/MS spectra that are used to query this database. As indicated in the first description of CFM-ID (34), this means users can predict MS/MS spectra from a given compound structure (called ‘C2MS’ for Compound to MS). It also means that users can identify a compound structure from a given MS/MS spectrum (called ‘MS2C’ for MS to Compound). After its initial release, CFM-ID was further modified to include support for electron-ionization mass spectrometry or EI-MS (27) and then upgraded to support rule-based fragmentation of lipids and compound class identification. These latter upgrades were included in the release of CFM-ID 3.0 (30). Since its first introduction in 2015, more than 3 million queries have been processed by CFM-ID 1.0, 2.0 and 3.0, including almost equal numbers of C2MS and MS2C predictions.

While CFM-ID remains very popular, a number of its algorithms, its performance and its visual displays have become somewhat dated. This motivated us to start upgrading both the back-end and the front-end of the CFM-ID server. For instance, recent developments in machine learning along with the availability of an expanded MS/MS training set allowed us to substantially improve the performance of the fragmentation modeling in the CFM-ID algorithm (35). Such a significant improvement clearly had to be added the CFM-ID web server. Similarly, user requests and user feedback suggested that we should expand the rule-based fragmentation methods in CFM-ID to cover a wider range of ‘hard-to-fragment’ molecules such as lipids and flavonoids. Likewise, improvements to the user interface, expanding and updating the *in silico* and experimental MS/MS databases, enhancements to the MS/MS spectral displays and support for neutral loss spectral searching were all deemed to be essential to maintain CFM-ID’s relevance to the user community. This paper describes these upgrades and updates, by formally introducing the CFM-ID 4.0 web server. This paper also demonstrates how these enhancements have improved the overall accuracy and performance of CFM-ID relative to earlier versions and relative to competing software tools.

## GENERAL DESIGN AND OPERATION

The CFM-ID 4.0 web server offers three general functions: 1) predicting electrospray ionization quadrupole time-of-flight tandem mass spectra (ESI-QTOF-MS/MS) for chemical compounds over multiple CID energies (C2MS); 2) annotating ESI-QTOF-MS/MS spectra given the structure of the parent compound; and 3) identifying the chemical compound that produced the given centroided ESI-QTOF-MS/MS spectra (MS2C). These three functions are listed on the CFM-ID home page as: **Spectra Prediction**, **Peak Assignment**, and **Compound Identification**.

CFM-ID 4.0’s **Spectra Prediction** utility performs the C2MS operation. To use this function, a user must enter the structure of a neutral compound using either SMILES (17) or InChI (18) format. They must also select the desired spectral type (only ESI is offered in version 4.0, while EI is still available in version 3.0), the ion mode (positive or negative), and the adduct type (the parent ion adduct, usually M + H if in the positive mode). After entering these data and pressing the **Submit** button, the CFM-ID server then generates *in-silico* product ion MS/MS spectra for three different CID energies (10 eV, 20 eV and 40 eV). The output of the **Spectra Prediction** function is presented in two different formats: (1) a human-readable, downloadable text file containing the predicted MS/MS spectrum, and (2) an interactive image of the predicted MS/MS spectrum. Each displayed peak in the interactive spectral display includes a high precision *m/z* value, a relative intensity value, and an image of the most likely associated fragment ion structure. This information can be viewed by mousing-over each spectral peak. In addition to generating a predicted *in-silico* MS/MS spectrum, CFM-ID 4.0 will also retrieve the experimentally measured MS/MS spectrum, if such a spectrum exists in the CFM-ID experimental spectral library. This experimental MS/MS spectral library contains both internally collected and externally collected MS/MS spectra provided by MoNA (7), MassBank EU/Japan (8), GNPS (9) and others.

CFM-ID 4.0’s **Peak Assignment** utility is designed to annotate and explain each peak in a submitted experimental MS/MS spectrum along with the corresponding (submitted) structure. This utility is intended to help improve the explainability of product ion MS/MS spectra and has been widely used as a teaching tool. For a given MS/MS spectrum and the corresponding (known) chemical structure, the **Peak Assignment** tool attempts to assign a possible fragment ion to each peak in the MS/MS spectrum. To use the **Peak Assignment** utility, a user must submit the known compound structure and a corresponding list of *m/z* peaks from an ESI-MS/MS experiment. Users must also select the corresponding charge type, adduct type, and mass tolerance value. The mass tolerance value (default to 10 ppm) is the tolerance used to match the observed MS/MS peaks to the predicted fragment ions calculated by CFM-ID 4.0. As with the **Spectra Prediction** utility, the output for **Peak Assignment** is displayed in a color-coded mass spectrogram, where annotated peaks are marked in red and unannotated peaks (if any) in blue. In addition to generating an interactively viewable MS/MS spectrum, where fragment ion structures can be viewed by mousing-over a given peak, the user can also download an additional text file. This file contains the annotated peak list and the corresponding SMILES strings for the identified ion fragments.

CFM-ID 4.0’s **Compound Identification** utility supports its MS2C operations. In particular, it allows users to identify metabolites from one or more user-supplied experimental MS/MS spectra. CFM-ID 4.0 offers users two options: 1) Regular **Compound Identification** via product ion MS/MS spectra and 2) compound identification via **Neutral Loss Search** (36). The Regular **Compound Identification** option requires the user to upload an experimentally measured product ion MS/MS spectrum of a (reasonably) pure compound collected at one or more specified collision energies: low (10 eV), medium (20 eV), and/or high (40 eV) – either entered directly in text boxes or uploaded as files. Users must also supply the desired spectral type (only ESI is offered in version 4.0), the ion mode (positive or negative), the adduct type (the parent ion adduct), the parent ion mass (measured from the ion selection filter to collect the MS/MS spectrum), the candidate mass tolerance, the scoring function (to rank the spectral matches), the number of results to be viewed and the mass tolerance for peak matching in the spectral display (default to 10 ppm). Users must also select from a set of 18 databases carefully curated databases containing experimental and/or *in silico*-predicted MS/MS spectra along with their corresponding compound structures. Once these data are submitted, by pressing the **Submit** button, it typically takes a less than a minute for the CFM-ID server to return a sorted list of possible compound structures. The amount of time taken depends on the number and size of the spectral databases being searched. While compound structures can be identified with only a single MS/MS spectrum collected at a single collision energy level, providing spectra at two or more energy levels will certainly improve the identification accuracy. Users can specify which of three methods for scoring the similarity between observed versus predicted MS/MS spectra to use: 1) the Dice score, 2) the dot product score, and 3) the dot product + Metadata score. Details of the Dice score and dot product ranking methods can be found in reference (27), while details about the dot product + Metadata scoring method are available in reference (30). The CFM-ID 4.0 website provides two example spectra (Example #1 and Example #2) for users to test.

**Figure 1.**
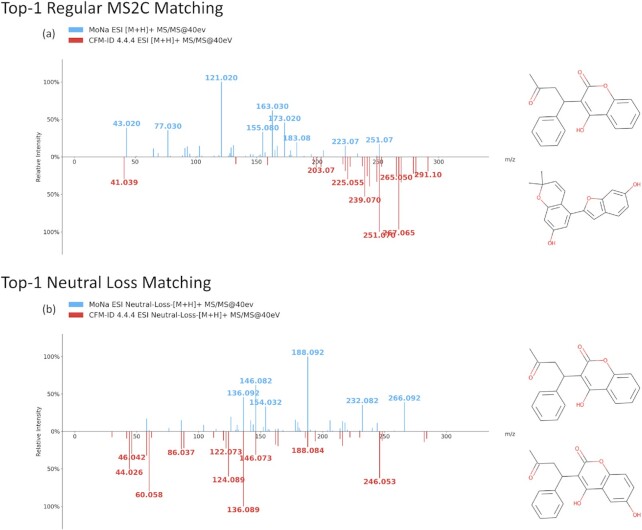
An example illustrating the utility of the **Neutral Loss Search** for the spectra-to-compound (MS2C) identification task where the target structure and spectrum are not available in any CFM-ID database. For this example, the query spectrum and query compound was Warfarin and the database searched was the *in silico* CFM-ID spectral database (with Warfarin removed from the database). a) Shows the spectral comparison between the query product ion MS/MS spectrum (warfarin) and its top matched candidate (moracin E) using the regular **Compound Identification** option. b) Shows the spectral comparison between the query neutral loss spectrum (automatically calculated from the product ion MS/MS spectrum) and its top matched candidate ((R)-6-hydroxywarfarin) using the **Neutral Loss Search** option. As shown here, the **Neutral Loss Search** can find a structurally similar candidate that cannot be found by a simple product ion search using the **Compound Identification** option.

In contrast to the regular **Compound Identification**, the **Neutral Loss Search** can be used to identify or partially characterize novel compounds that are not in CFM-ID’s product ion MS/MS spectral databases. Neutral loss spectra are subtractive or theoretical spectra generated from a conventional product ion MS/MS spectrum by determining the mass differences between the precursor ion *m/z* and each of the other peaks in the product ion spectrum. By generating MS/MS spectra displaying *m/z* differences rather than actual *m/z* values, it is possible to identify characteristic fragment ions or fragment substructures. As a result, neutral loss MS/MS spectral searching is ideal for identifying substructures from larger, conjugated molecules or for identifying molecules that differ from each other by the addition (or loss) of a smaller ‘substructure’ such as a water molecule, an ammonia molecule, a phosphate group, a sugar group or some other minor chemical modification. Normally small *m/z* shifts arising from minor chemical modifications make it impossible for conventional MS/MS spectral searching operations to identify chemically modified or chemically similar molecules. On the other hand, neutral loss searching allows these minor modifications to be ignored during the spectral searching process. In this way CFM-ID’s **Neutral Loss Search** allows users to identify chemical compounds that are chemically similar to chemical compounds in the CFM-ID 4.0 spectral databases but which have no actual structure or actual product ion MS/MS spectra in the CFM-ID 4.0 databases. An illustrative example of a **Neutral Loss Search** using CFM-ID 4.0 is shown in Figure 1. As shown here, a regular product ion **Compound Identification** search with an experimentally measured MS/MS spectrum of warfarin (where the MS/MS spectrum of warfarin has been deliberately removed from the selected CFM-ID database) yields no significant, or chemically similar matches. On the other hand, a **Neutral Loss Search** of the same compound that uses the automatically calculated neutral loss spectrum of warfarin against CFM-ID’s neutral loss MS/MS database identifies one molecule that is almost chemically identical to warfarin as its top hit. Similar to other interactive spectral viewers in CFM-ID, mousing over the neutral loss ions allows users to identify the structures of many of the substituent ions. These high scoring hits and the identification of key neutral loss ions off the opportunity for users to determine the approximate structure of hitherto unknown compounds or compounds not in the CFM-ID spectral databases.

To identity or partially identify a structure via the **Neutral Loss Search** (37), users must upload the experimentally measured MS/MS spectra (at one or more collision energies), select the preferred candidate databases, and supply other information needed by the regular **Compound Identification** search. For the **Neutral Loss Search** option, the user-specified ranking function is limited to only the Dice Score. In performing the **Neutral Loss Search**, the CFM-ID web server will first compute the neutral loss spectra from the user's supplied product ion MS/MS spectra and then perform a spectral match between these neutral loss spectra and all calculated neutral loss MS/MS spectra in the selected databases. This typically takes about a minute (depending on the number of databases selected) for the server to complete the calculation and to sort the hits. The **Neutral Loss Search** provides two example spectra (Example #1 and #2) for users to try.

**Figure 2.**
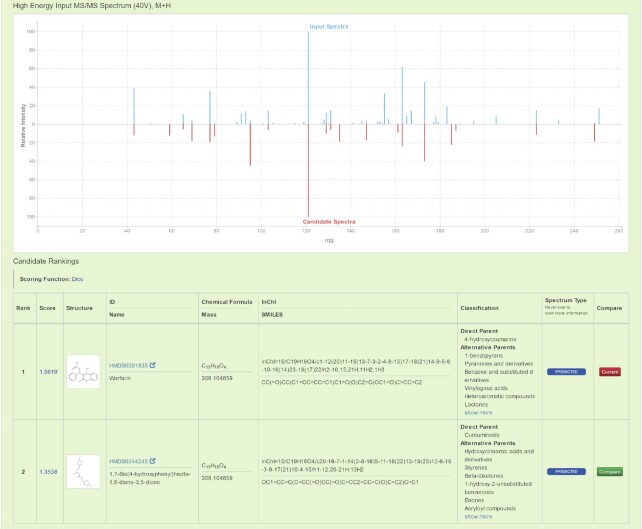
An example of the output from a regular **Compound Identification** query using the experimentally acquired product ion MS/MS spectrum of Warfarin against the *in silico* CFM-ID spectral databases. The top half of the figure illustrates the mirrored MS/MS spectral plot between the experimental MS/MS spectrum (blue peaks), and the highest ranked candidate *in silico* MS/MS spectrum (red peaks). A table with the detailed candidate scores, structures and spectral links is presented on the bottom half of the page.

The output of both the **Neutral Loss Search** and regular **Compound Identification** function is presented in two parts: an MS/MS mirror plot for comparing spectra and a tabular list of ranked compounds. An example of such an output can be found in Figure 2, where the user-supplied product ion MS/MS spectrum is displayed in the top half (in blue) and the matching MS/MS spectrum found from the database search is presented in the bottom half of the mirror plot (in red). As with the other MS spectral displays generated by CFM-ID, moving the cursor over each peak in the predicted/matched spectrum will also trigger the display the structure of the CFM-ID predicted fragments or the predicted neutral losses. The list of top-ranked compounds/spectra is located under the spectral mirror plot, with each row in the scrollable table consisting of the ranking score, a structure image, the chemical formula, the molecular weight and the ClassyFire (38) chemical classification results. Using this scrollable table, users can select any spectrum from any of the listed compounds to compare with their queried MS/MS spectra. Clicking on a different compound from the table will automatically replace the mirror plot(s) with the corresponding MS/MS spectra. CFM-ID 4.0 has added significantly more information about each candidate and structure to help users better examine and evaluate their **Compound Identification** results.

## FRONT-END IMPROVEMENTS

Some of the most notable improvements to CFM-ID 4.0 have been made to its user interface. In particular, a number of improvements were made including: modernizing the web server's appearance, simplifying the workflow, providing a more comprehensive and easier-to-understand **Help** section, and most importantly, offering a better interactive display of the MS/MS spectra. More specifically, we redesigned CFM-ID 4.0’s **Home Page**, so that it now shares the same layout and styling with our other popular databases and web servers such as HMDB 5.0 (6) and NP-MRD (39). This re-worked home page is more self-explanatory and straightforward than the previous design. We also updated the **Help** section with a more visual step-by-step guide for each of the three utilities. In contrast to the text-only **Help** section offered in previous versions, CFM-ID 4.0’s new **Help** section is easier to understand with annotated screenshots provided at each step. In addition to the updated **Help** section, extra assistance has been made available through question mark icons and pop-up explanations. Perhaps the most important front-end improvement for CFM-ID 4.0 has been the updated **MS Spectral Viewer** featured in the **Compound Identification** utility. Rather than displaying two MS/MS spectra separately, this updated viewer offers a mirrored view of the two MS/MS spectra with a shared x-axis. Since all peaks are naturally aligned by their *m/z* peaks, visually comparing and identifying matching peaks is now much more accessible through this improved visualization tool.

## BACK-END IMPROVEMENTS

While CFM-ID 4.0’s improved front-end now offers a better user experience, improvements to the back-end and many of its underlying algorithms have significantly improved CFM-ID’s overall performance and accuracy. There are two categories of back-end improvements. First, we updated the MS/MS spectral prediction tool with the latest version of the CFM-ID algorithm (34). For any C2MS task, such as **Spectra Prediction** or **Peak Assignment**, the CFM-ID web server will first attempt to compute the MS/MS spectra via a rule-based algorithm called MSRB (Mass Spectra Rule-Based). While the MSRB algorithm is faster than the machine learned algorithm, it can only compute MS/MS spectra for compounds from a relatively small set of chemical classes, including lipids, polyphenols, acylcarnitines and acylglycines (see (34) for the complete list of MSRB supported chemicals). If the MSRB algorithm cannot compute an MS/MS spectrum, the CFM-ID 4.0 webserver uses the machine-learned MS/MS predictor (called MSML) to predict MS/MS spectra. Both [M + H]+ and [M-H]- adduct types are fully supported for spectrum prediction tasks, while other adduct types are only partially supported. In cases where MS/MS spectra for a specific adduct type cannot be computed, it is assumed that those adducts would only be present for the precursor ion and not for any of the daughter ions. Obviously rare exceptions, such as triacylglycerol sodium adducts that generate sodium ion fragments, can occur. Nevertheless, based on this assumption, the CFM-ID 4.0 web server will return a [M + H]+ spectrum (or an [M-H]- spectrum depending on the charge type) with an extra peak at the calculated precursor adduct *m/z* value. The CFM-ID 4.0 web server uses the same noise removal setting as previous versions of CFM-ID.

## EVALUATION

**Figure 3.**
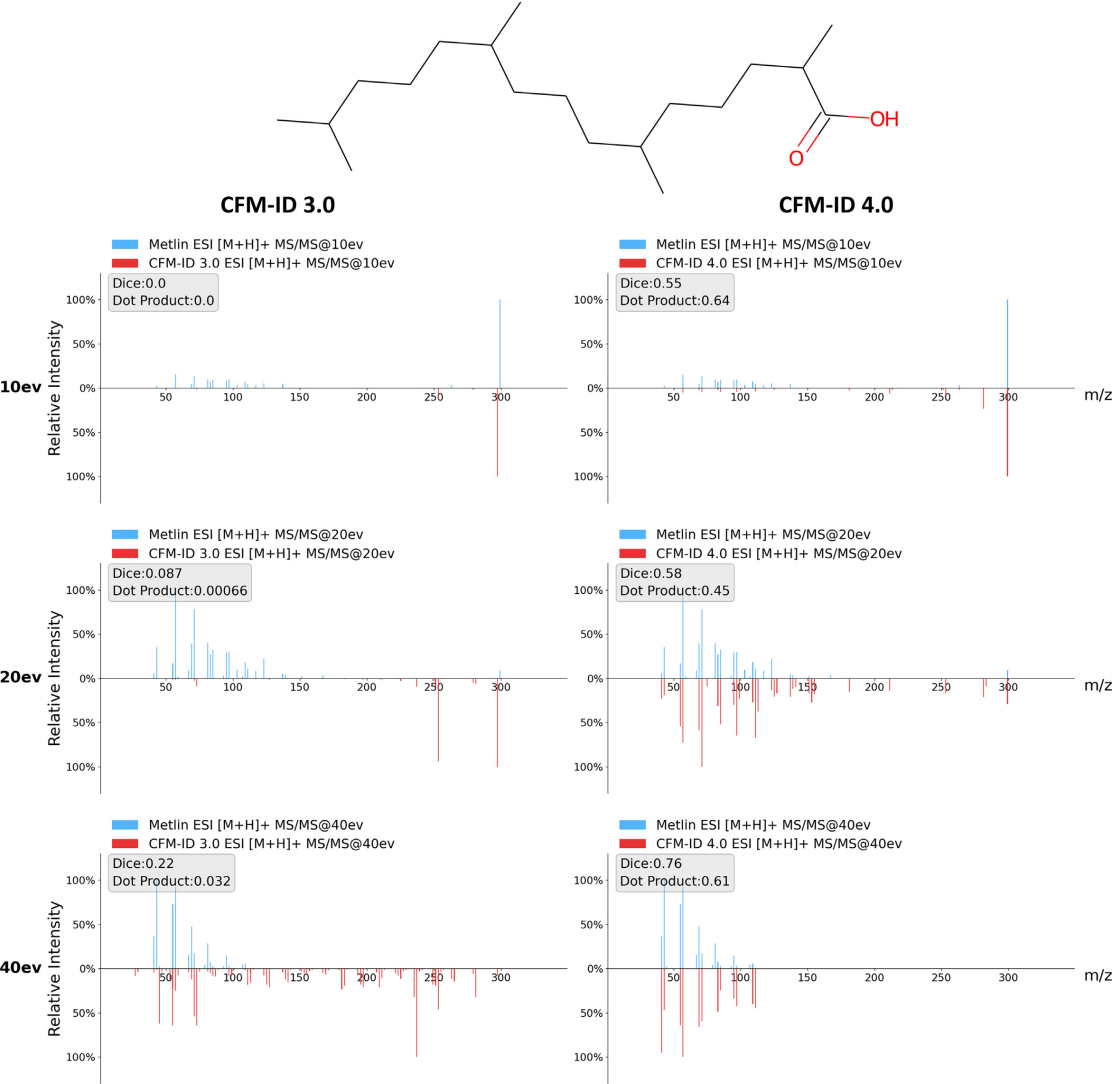
A comparison of the quality of CFM-ID 4.0 predicted MS/MS spectra versus CFM-ID 3.0 predicted MS/MS spectra. This figure compares the predicted ESI-MS/MS [M-H]- spectra of pristanic acid as predicted by CFM-ID 3.0 (on the left) and CFM-ID 4.0 (on the right) at different collision energies. The actual experimental MS/MS spectra for this compound are displayed on the top (blue peaks) while the predicted *in-silico* MS/MS spectra (red peaks) are displayed on the bottom of the mirror plots.

The changes introduced to CFM-ID 4.0 necessitated a careful evaluation of its prediction performance to ensure that these improvements were robust and significant. As described in (34), we performed 10-fold cross-validation for the [M + H]+ and [M-H]- C2MS operation (i.e. the **Spectra Prediction** utility) to assess its prediction performance. Compared to the CFM-ID 2.0 and the CFM-ID 3.0 web servers, CFM-ID 4.0 was able to predict MS/MS spectral significantly more accurately. As also discussed in (34), the predicted *in-silico* MS/MS spectra and experimentally collected MS/MS spectra, CFM-ID 4.0's prediction achieved an average (over multiple collision energies) dot product score of 0.38 and 0.35 for [M + H] + and [M-H]- spectra, respectively. This corresponds to a ∼26% and a ∼21% performance gain compared to CFM-ID 3.0. Figure 3 provides an example comparing the predicted MS/MS spectra from CFM-ID 3.0 to those generated by CFM-ID 4.0 along with their corresponding experimentally collected QTOF-MS/MS spectra. Specifically, this figure compares the predicted (and experimental) ESI-MS/MS [M-H]- product ion spectra of pristanic acid between CFM-ID 3.0 and CFM-ID 4.0. These images show that the MS/MS spectra predicted by CFM-ID 4.0 are much more similar to the experimentally collected MS/MS spectra, and they also have higher Dice and dot product scores. Furthermore, the CFM-ID 4.0 predicted MS/MS spectra are far less noisy than the predicted MS/MS spectra generated by CFM-ID 3.0.

We also tested CFM-ID 4.0 on the CASMI 2016 (2) Category-3 dataset for the C2MS task (i.e. **Compound Identification**). As described in (35), the CFM-ID 4.0 algorithm managed to identify 162 chemical compounds from experimentally collected Orbitrap MS/MS spectra out of 204 total testing cases. This result surpassed the performance of a number of other MS/MS identification tools such as MS-FINDER (40), MetFrag (25) and SIRIUS 4 (24). It is particularly notable that this result was achieved even though CFM-ID 4.0 was trained on QTOF-MS/MS spectra instead of Orbitrap MS/MS spectra. As a general rule, for the same molecule, QTOF CID MS/MS spectra and Orbitrap HCD (higher-energy collisional dissociation) MS/MS spectra collected at similar collision energies share many of the same fragment ions. There are often some differences in intensities for certain m/z fragments, and HCD spectra typically have more unique *m/z* fragments than their CID counterparts. Because CFM-ID was trained exclusively on QTOF CID data, its predicted MS/MS spectra are more to QTOF spectra than Orbitrap spectra. Nevertheless, QTOF-CID spectra predicted by CFM-ID can be used to accurately identify chemical compounds using Orbitrap HCD spectra. This can be done by determining the equivalent CID energy of a given Orbitrap HCD spectrum from its normalized collision energy (NCE) value, then comparing this Orbitrap HCD spectrum with the CFM-ID-predicted spectrum with a CID energy that is closest to the NCE (41). This was the procedure used for evaluating CFM-ID 4.0’s performance on the CASMI 2016 challenge. To further benchmark CFM-ID 4.0’s performance, we conducted an additional chemical compound identification evaluation using MS/MS spectra for 401 chemical compounds with experimentally collected [M + H]+ MS/MS spectra and 237 chemical compounds with experimentally collected [M-H]- MS/MS spectra from the HMDB 5.0 database. Every chemical compound in this test set had experimental MS/MS spectra collected at 10, 20, 40 eV on an ESI QTOF-MS/MS instrument as indicated from their original entry data in the MoNA (7) and NIST 20 (12) databases. This evaluation involved two experiments: one used the ∼250,000 chemical compounds in HMDB 5.0 (and their predicted MS/MS spectra) as a candidate library, the other used a portion of PubChem (∼ 2.1 million chemical compounds and their predicted MS/MS spectra) as the candidate library. Note that each target chemical compound used in this test had at least three candidate chemical compounds (with the same parent ion mass) with the average number of candidates being > 11 in the HMDB data set and > 2500 in the PubChem data set. To ensure the test was fair, we excluded all chemical compounds from the CFM-ID 4.0 training dataset and included only *in-silico* predicted MS/MS spectra (i.e. we excluded any experimentally collected MS/MS data from CFM-ID’s database). As noted previously in (30), including any experimental MS/MS spectra of the query compound in the candidate MS/MS spectral library will almost always guarantee a correct identification. Therefore, this benchmark provides a lower bound of what the CFM-ID 4.0 web server is capable of doing. Table 1 shows that CFM-ID 4.0 was able to correctly identify 48.1% (in the positive mode) and 38.4% (in the negative mode) of the query compounds when searching the HMDB 5.0 spectral library. Furthermore, 95.8% and 93.7% of the query compounds can be found within the top 10 ranked candidates for the positive ion mode and negative ion mode respectively. When using PubChem (42) (which is a much larger database – averaging 4096 candidates and 1624 candidates for [M + H]+ and [M-H]- respectively) as the candidate library, the identification accuracy was somewhat lower. However, CFM-ID still managed to identify 7.0% and 6.3% of the query compounds from their given MS/MS spectra using the positive and negative ion modes, respectively.

**Table 1.** Summary of Compound Identification results using the CFM-ID 4.0 web server and different scoring options. Searches were performed against the HMDB 5.0 database (∼250,000 compounds) and a subset of PubChem (∼2.1 million compounds). The median number of candidates with the same parent ion mass is listed under ‘Candidate Median’. The Rank indicates the position in the list of spectral hits where the correct compound was found. The percentage of compounds with the correct molecular formula based on the top ranked hit (even with the incorrect structure) is given in ‘% Correct formula for the first hit’

		HMDB 5.0		PubChem	
		[M + H]+	[M-H]-	[M + H]+	[M-H]-
	Candidate Median	9	10	2764	1624
CFM-ID 4.0 (dot product)	Rank = 1	48.10%	38.40%	7.01%	6.30%
	Rank $ \le$ 5	89.50%	82.30%	20.70%	19.50%
	Rank $ \le$ 10	95.80%	93.70%	29.94%	30.10%
	% Correct formula for the first hit	98.20%	97.80%	90.76%	82.70%
CFM-ID 4.0 (Dice)	Rank = 1	48.13%	32.49%	5.41%	5.50%
	Rank $ \le$ 5	87.53%	82.70%	16.24%	19.50%
	Rank $ \le$ 10	95.01%	93.67%	24.20%	27.90%
	% Correct formula for the first hit	99.25%	98.73%	93.31%	84.90%

Although CFM-ID 4.0 is by no means perfect in identifying compounds purely from their MS/MS spectra, it is almost perfect at determining the correct chemical formula. As shown in Table 1, when we queried product ion MS/MS data against the HMDB 5.0, the top-ranked chemical compound was found to have the correct molecular formula in more than 98% of the cases. This was regardless of the ion detection mode. This performance drops to 85% when querying the PubChem database. This is because the PubChem database had more than 200 times more candidates than the HMDB on average. In another performance evaluation test, we compared CFM-ID 4.0’s **Compound Identification** (product ion MS/MS) performance to SIRIUS 4 (24) using HMDB 5.0 as the chemical compound library. Table 2 shows that CFM-ID 4.0 (using its generated set of *in silico* HMDB 5.0 spectra) outperformed SIRIUS 4 in terms of chemical compound identification accuracy for the positive ion mode, while SIRIUS 4 showed a slight advantage in the negative ion mode (for the top ranked hits). Interestingly, SIRIUS 4 could only identify chemical compounds from a given MS/MS spectrum in 93 out of 108 cases. There were 15 cases where no chemical structure was produced, and 4 out of these 15 cases proposed no chemical formula.

**Table 2.** Summary of Compound Identification results and molecular formula determination results between SIRIUS4 and the CFM-ID 4.0 web server using different scoring options (for CFM-ID). Searches were performed against the HMDB 5.0 database (∼250,000 chemical compounds). The median number of candidates with the same parent ion mass is listed under ‘Candidate Median’. The Rank indicates the position in the list of spectral hits where the correct compound was found. The percentage of chemical compounds with the correct molecular formula based on the top ranked hit (even with the incorrect structure) is given in ‘% Correct formula for the first hit’

		[M + H]+	[M-H]−
	Candidate Median	11	12
CFM-ID 4.0 (dot product)	Rank = 1	41.51%	31.58%
	Rank $ \le$ 5	79.25%	78.95%
	Rank $ \le$ 10	92.45%	91.23%
	% Correct formula for the first hit	98.11%	100.00%
CFM-ID 4.0 (Dice)	Rank = 1	37.74%	28.57%
	Rank $ \le$ 5	79.25%	83.93%
	Rank $ \le$ 10	86.79%	92.86%
	% Correct formula for the first hit	98.11%	100.00%
SIRIUS 4	Rank = 1	19.23%	35.71%
	Rank $ \le$ 5	56.69%	69.64%
	Rank $ \le$ 10	67.31%	75.00%
	% Correct formula for the first hit	96.42%	82.69%

## IMPLEMENTATION

The CFM-ID 4.0 web server is organized into two components: 1) the web layer that serves all the web pages and handles data storage and 2) the computational core, which handles all of the spectral predictions and calculations. The web layer was developed using the Ruby on Rails framework (version 5). Ruby on Rails is a development system that employs the Model-View-Controller (MVC) concept, where models respond and interact with the data, views create the interface to show and interact with the data, and controllers connect the user to the views. This framework allowed the CFM-ID 4.0 programming team to rapidly develop, prototype and test all CFM-ID’s web modules and page views. MySQL and Redis were used for the back-end and HTML, CSS, JavaScript, and the D3.js library were used for the front-end. The computational core consists of several specialized CFM-ID 4.0 algorithms developed in C++ and Java, including the machine-learned models and the rule-based extension implementations (CFM-ID MSML 4.4.5 and CFM-ID MSRB 1.1.13). The machine-learned models deployed on the CFM-ID 4.0 web server were all derived from the recently published updates to the CFM-ID algorithm (34). Each component of the CFM-ID 4.0 server system is fully containerized via Docker, which combined with its two-tier design ensures good scalability and stability. Dockerizing the system also gave the team the flexibility to develop and test each component of the server system individually. CFM-ID 4.0’s computational core image is also provided as a freely downloadable file via hub.docker (https://hub.docker.com/r/wishartlab/cfmid). This docker image only contains the core functionality of CFM-ID and does not include any pre-computed MS/MS spectral data. More information is available in CFM-ID’s **Help** section about this core image. However, the downloadable version of CFM-ID 4.0 enables large-scale computation or the processing of sensitive data, which cannot easily be supported on a publicly accessible server. The CFM-ID server is hosted on a quad-core (Intel Xeon 8175M) virtual server with 8G of RAM. The current configuration has a three minute time-out on MS/MS spectral prediction tasks. The amount of time required to perform a spectral prediction is largely dependent on the molecular weight (MW) and the number of bonds in the molecule. Typically, a molecule with a MW < 1000 Da will take less than one minute to predict.

## CONCLUSION AND FUTURE PLANS

The CFM-ID 4.0 web server offers a suite of utilities to facilitate automated MS/MS spectral prediction (C2MS), spectral annotation and chemical compound identification (MS2C). Compared to previous versions, the CFM-ID 4.0 web server is more user-friendly, more accurate and more informative. In particular, CFM-ID 4.0’s functional improvements include an improved user interface, a new suite of neutral loss searches and neutral loss annotations, improved spectral displays (mirror plots), more informative data tables, improved prediction capabilities, enhanced documentation and greater user-friendliness. CFM-ID 4.0’s C2MS performance has been thoroughly benchmarked and proven to be significantly better than previous versions (34). Its MS2C performance has been tested on multiple MS/MS datasets against many different candidate databases both here and elsewhere (34). These results also show that CFM-ID 4.0 not only performs well with QTOF MS/MS data, but also outperformed all other tools in the CASMI 2016 Category-3 challenge test even though CASMI used only Orbitrap MS/MS spectra, not QTOF MS/MS. Most importantly, CFM-ID 4.0’s MS2C results are much more explainable and queryable than the previous CFM-ID versions as well as other popular tools such as SIRIUS4 (24) and MetFrag (25). With more accurate *in-silico* MS/MS spectra, a greatly expanded CFM-ID spectra library, and the introduction of a **Neutral Loss Search** feature, we believe the CFM-ID 4.0 web server will be much more useful to users wishing to perform MS2C tasks for identifying known structures or those wishing to identify completely novel structures. While the improvements to both front-end and back-end are significant, we are still planning to implement several extensions to the CFM-ID 4.0 web server over the coming year. In particular, we expect to re-introduce a much-improved EI-MS spectra prediction module and a significantly improved EI-MS compound identification module (for GC-MS-based metabolomics) that will support user input of experimentally measured retention indices and EI-MS spectra. We also plan to extend the next version of CFM-ID to support Orbitrap MS/MS spectral predictions. These additions will likely be introduced in later 2022 or early 2023. Overall, we believe the improvements already described here along with the planned improvements to the server's functionality will make CFM-ID 4.0 much more useful to the analytical chemistry community and should make small molecule identification easier, faster and more precise.

## References

[1] Alonso A. , Marsal S., Julia A. Analytical methods in untargeted metabolomics: state of the art in 2015. Front. Bioeng. Biotechnol. 2015; 3:23. 25798438 10.3389/fbioe.2015.00023 PMC4350445

[2] Cebo M. , Schlotterbeck J., Gawaz M., Chatterjee M., Lämmerhofer M. Simultaneous targeted and untargeted UHPLC-ESI-MS/MS method with data-independent acquisition for quantification and profiling of (oxidized) fatty acids released upon platelet activation by thrombin. Anal. Chim. Acta. 2020; 1094:57–69. 31761048 10.1016/j.aca.2019.10.005

[3] Vitale C.M. , Price E.J., Miller G.W., David A., Antignac J.-P., Barouki R., Klánová J. Analytical strategies for chemical exposomics: exploring limits and feasibility. Exposome. 2021; 1:osab003.

[4] Strayer K.E. , Antonides H.M., Juhascik M.P., Daniulaityte R., Sizemore I.E. LC-MS/MS-based method for the multiplex detection of 24 fentanyl analogues and metabolites in whole blood at sub ng mL–1 concentrations. ACS Omega. 2018; 3:514–523. 29399650 10.1021/acsomega.7b01536 PMC5793031

[5] Ayala-Cabrera J.F. , Santos F.J., Moyano E. Recent advances in analytical methodologies based on mass spectrometry for the environmental analysis of halogenated organic contaminants. Trends Environ. Anal. Chem. 2021; 30:e00122.

[6] Wishart D.S. , Guo A., Oler E., Wang F., Anjum A., Peters H., Dizon R., Sayeeda Z., Tian S., Lee B.L. et al. HMDB 5.0: the human metabolome database for 2022. Nucleic Acids Res. 2021; 50:D622–D631. 10.1093/nar/gkab1062 PMC8728138 34986597

[7] Slobodnik J. , Hollender J., Schulze T., Schymanski E.L., Brack W. Establish data infrastructure to compile and exchange environmental screening data on a european scale. Environ. Sci. Eur. 2019; 31:65.

[8] Horai H. , Arita M., Kanaya S., Nihei Y., Ikeda T., Suwa K., Ojima Y., Tanaka K., Tanaka S., Aoshima K. et al. MassBank: a public repository for sharing mass spectral data for life sciences. J. Mass Spectrom. 2010; 45:703–714. 20623627 10.1002/jms.1777

[9] Wang M. , Carver J.J., Phelan V.V., Sanchez L.M., Garg N., Peng Y., Nguyen D.D., Watrous J., Kapono C.A., Luzzatto-Knaan T. et al. Sharing and community curation of mass spectrometry data with global natural products social molecular networking. Nat. Biotechnol. 2016; 34:828–837. 27504778 10.1038/nbt.3597 PMC5321674

[10] Stephen S. NIST/EPA/NIH mass spectral library with search program data version: NIST v14 mass spectrometry data center national institute of standards and technology. 2014;

[11] Stephen S. NIST/EPA/NIH mass spectral library with search program data version: NIST v17 mass spectrometry data center national institute of standards and technology. 2017;

[12] Stephen S. NIST/EPA/NIH mass spectral library with search program data version: NIST v20 mass spectrometry data center national institute of standards and technology. 2020;

[13] Smith C.A. , O’maille G., Want E.J., Qin C., Trauger S.A., Brandon T.R., Custodio D.E., Abagyan R., Siuzdak G. METLIN : a metabolite mass spectral database. Ther. Drug Monit. 2005; 27:747–751. 16404815 10.1097/01.ftd.0000179845.53213.39

[14] Guijas C. , Montenegro-Burke J.R., Domingo-Almenara X., Palermo A., Warth B., Hermann G., Koellensperger G., Huan T., Uritboonthai W., Aisporna A.E. et al. METLIN: a technology platform for identifying knowns and unknowns. Anal. Chem. 2018; 90:3156–3164. 29381867 10.1021/acs.analchem.7b04424 PMC5933435

[15] Mushtaq S. , Abbasi B.H., Uzair B., Abbasi R. Natural products as reservoirs of novel therapeutic agents. EXCLI J. 2018; 17:420–451. 29805348 10.17179/excli2018-1174 PMC5962900

[16] Sorokina M. , Merseburger P., Rajan K., Yirik M.A., Steinbeck C. COCONUT online: collection of open natural products database. J. Cheminform. 2021; 13:2. 33423696 10.1186/s13321-020-00478-9 PMC7798278

[17] Richard A.M. , Williams C.R. Distributed structure-searchable toxicity (DSSTox) database. Mutat. Res. 2022; 499:27–52. 10.1016/s0027-5107(01)00289-5 11804603

[18] Dionisio K.L. , Phillips K., Price P.S., Grulke C.M., Williams A., Biryol D., Hong T., Isaacs K.K. The chemical and products database, a resource for exposure-relevant data on chemicals in consumer products. Sci. Data. 2017; 5:180125. 10.1038/sdata.2018.125 PMC6038847 29989593

[19] da Silva R.R. , Dorrestein P.C., Quinn R.A. Illuminating the dark matter in metabolomics. Proc. Natl. Acad. Sci. USA. 2015; 112:12549–12550. 26430243 10.1073/pnas.1516878112 PMC4611607

[20] Peisl B.Y.L. , Schymanski E.L., Wilmes P. Dark matter in host-microbiome metabolomics: tackling the unknowns–A review. Anal. Chim. Acta. 2018; 1037:13–27. 30292286 10.1016/j.aca.2017.12.034

[21] Heinonen M. , Shen H., Zamboni N., Rousu J. Metabolite identification and molecular fingerprint prediction through machine learning. Bioinformatics. 2012; 28:2333–2341. 22815355 10.1093/bioinformatics/bts437

[22] Shen H. , Zamboni N., Heinonen M., Rousu J. Metabolite identification through machine learning— tackling casmi challenge using fingerid. Metabolites. 2013; 3:484–505. 24958002 10.3390/metabo3020484 PMC3901273

[23] Shen H. , Dührkop K., Böcker S., Rousu J. Metabolite identification through multiple kernel learning on fragmentation trees. Bioinformatics. 2014; 30:i157–i164. 24931979 10.1093/bioinformatics/btu275 PMC4058957

[24] Dührkop K. , Fleischauer M., Ludwig M., Aksenov A.A., Melnik A.V., Meusel M., Dorrestein P.C., Rousu J., Böcker S. SIRIUS 4: a rapid tool for turning tandem mass spectra into metabolite structure information. Nat. Methods. 2019; 16:299–302. 30886413 10.1038/s41592-019-0344-8

[25] Ruttkies C. , Schymanski E.L., Wolf S., Hollender J., Neumann S. MetFrag relaunched: incorporating strategies beyond in silico fragmentation. J. Cheminform. 2016; 8:3. 26834843 10.1186/s13321-016-0115-9 PMC4732001

[26] Kind T. , Liu K.-H., Lee D.Y., DeFelice B., Meissen J.K., Fiehn O. LipidBlast in silico tandem mass spectrometry database for lipid identification. Nat. Methods. 2013; 10:755–758. 23817071 10.1038/nmeth.2551 PMC3731409

[27] Allen F. , Pon A., Greiner R., Wishart D.S. Computational prediction of electron ionization mass spectra to assist in GC/MS compound identification. Anal. Chem. 2016; 88:7689–7697. 27381172 10.1021/acs.analchem.6b01622

[28] Allen F. , Greiner R., Wishart D.S. Competitive fragmentation modeling of ESI-MS/MS spectra for putative metabolite identification. Metabolomics. 2015; 11:98–110.

[29] Wei J.N. , Belanger D., Adams R.P., Sculley D Rapid prediction of electron-ionization mass spectrometry using neural networks. ACS Cent. Sci. 2019; 5:700–708. 31041390 10.1021/acscentsci.9b00085 PMC6487538

[30] Djoumbou-Feunang Y. , Pon A., Karu N., Zheng J., Li C., Arndt D., Gautam M., Allen F., Wishart D.S. CFM-ID 3.0: significantly improved ESI-MS/MS prediction and compound identification. Metabolites. 2019; 9:72. 10.3390/metabo9040072 PMC6523630 31013937

[31] Laponogov I. , Sadawi N., Galea D., Mirnezami R., Veselkov K.A. ChemDistiller: an engine for metabolite annotation in mass spectrometry. Bioinformatics. 2018; 34:2096–2102. 29447341 10.1093/bioinformatics/bty080 PMC9881669

[32] Dührkop K. , Shen H., Meusel M., Rousu J., Böcker S. Searching molecular structure databases with tandem mass spectra using CSI:FingerID. Proc. Natl/Acad. Sci. USA. 2015; 112:12580–12585. 10.1073/pnas.1509788112 PMC4611636 26392543

[33] Creese A.J. , Cooper H.J. Liquid chromatography electron capture dissociation tandem mass spectrometry (LC-ECD-MS/MS) versus liquid chromatography collision-induced dissociation tandem mass spectrometry (LC-CID-MS/MS) for the identification of proteins. J. Am. Soc. Mass Spectrom. 2007; 18:891–897. 17350280 10.1016/j.jasms.2007.01.008 PMC2572008

[34] Allen F. , Pon A., Wilson M., Greiner R., Wishart D.S. CFM-ID: a web server for annotation, spectrum prediction and metabolite identification from tandem mass spectra. Nucleic Acids Res. 2014; 42:W94–W99. 24895432 10.1093/nar/gku436 PMC4086103

[35] Wang F. , Liigand J., Tian S., Arndt D., Greiner R., Wishart D.S. CFM-ID 4.0: more accurate ESI-MS/MS spectral prediction and compound identification. Anal. Chem. 2021; 93:11692–11700. 34403256 10.1021/acs.analchem.1c01465 PMC9064193

[36] Aisporna A. , Benton H.P., Chen A., Derks R.J.E., Galano J.M., Giera M., Siuzdak G. Neutral loss mass spectral data enhances molecular similarity analysis in METLIN. J. Am. Soc. Mass Spectrom. 2022; 33:530–534. 35174708 10.1021/jasms.1c00343 PMC10131246

[37] Moorthy A.S. , Wallace W.E., Kearsley A.J., Tchekhovskoi D.V., Stein S.E. Combining fragment-ion and neutral-loss matching during mass spectral library searching: a new general purpose algorithm applicable to illicit drug identification. Anal. Chem. 2017; 89:13261–13268. 29156120 10.1021/acs.analchem.7b03320 PMC5841953

[38] Djoumbou Feunang Y. , Eisner R., Knox C., Chepelev L., Hastings J., Owen G., Fahy E., Steinbeck C., Subramanian S., Bolton E. et al. ClassyFire: automated chemical classification with a comprehensive, computable taxonomy. J. Cheminform. 2016; 8:61. 27867422 10.1186/s13321-016-0174-y PMC5096306

[39] Wishart D.S. , Sayeeda Z., Budinski Z., Guo A., Lee B.L., Berjanskii M., Rout M., Peters H., Dizon R., Mah R. et al. NP-MRD: the natural products magnetic resonance database. Nucleic Acids Res. 2021; 50:D665–D677. 10.1093/nar/gkab1052 PMC8728158 34791429

[40] Vaniya A. , Samra S.N., Palazoglu M., Tsugawa H., Fiehn O. Using MS-FINDER for identifying 19 natural products in the CASMI 2016 contest. Phytochem. Lett. 2017; 21:306–312. 31576201 10.1016/j.phytol.2016.12.008 PMC6771281

[41] Szabó D. , Schlosser G., Vékey K., Drahos L., Révész Á. Collision energies on QTof and orbitrap instruments: how to make proteomics measurements comparable. J. Mass Spectrom. 2021; 56:e4693. 33277714 10.1002/jms.4693

[42] Bolton E.E. , Wang Y., Thiessen P.A., Bryant S.H. PubChem: integrated platform of small molecules and biological activities. Annu. Rep. Comput. Chem. 2008; 4:217–241.

